# Socioeconomic inequalities in the Pace of Aging

**DOI:** 10.18632/aging.204595

**Published:** 2023-03-10

**Authors:** Stephanie Schrempft, Silvia Stringhini

**Affiliations:** 1 Division of Primary Care, Unit of Population Epidemiology, Geneva University Hospitals, Switzerland; 2 Department of Health and Community Medicine, Faculty of Medicine, University of Geneva, Switzerland; 3 University Centre for General Medicine and Public Health, University of Lausanne, Switzerland

**Keywords:** socioeconomic inequalities, social mobility, Pace of Aging

Socioeconomic disadvantage is one of the most important predictors of morbidity and premature mortality [1]. Exposure to chronic social adversity, or exposure to social adversity during critical periods of development, is thought to trigger epigenetic processes that accelerate age-related physiological decline [2]. Childhood and adulthood socioeconomic disadvantage have been associated with dysregulation in multiple physiological systems [2], but there is a need for studies that link life course socioeconomic conditions and experiences of social adversity with longitudinal measures of age-related decline. The “Pace of Aging”, developed by the New Zealand-based Dunedin Study, is a longitudinal measure of aging based on within-individual changes in organ-system integrity markers [3]. Faster Pace of Aging is linked with declines in physical and cognitive functioning and brain aging, suggesting that intervention to slow Pace of Aging could extend healthspan. Previous research found that children who grew up in lower socioeconomic status homes exhibited faster Pace of Aging in midlife [4], but it is not known how life course socioeconomic conditions relate to the Pace of Aging, and whether the impact of adverse social exposures is reversible via upward social mobility.

**Figure 1:**
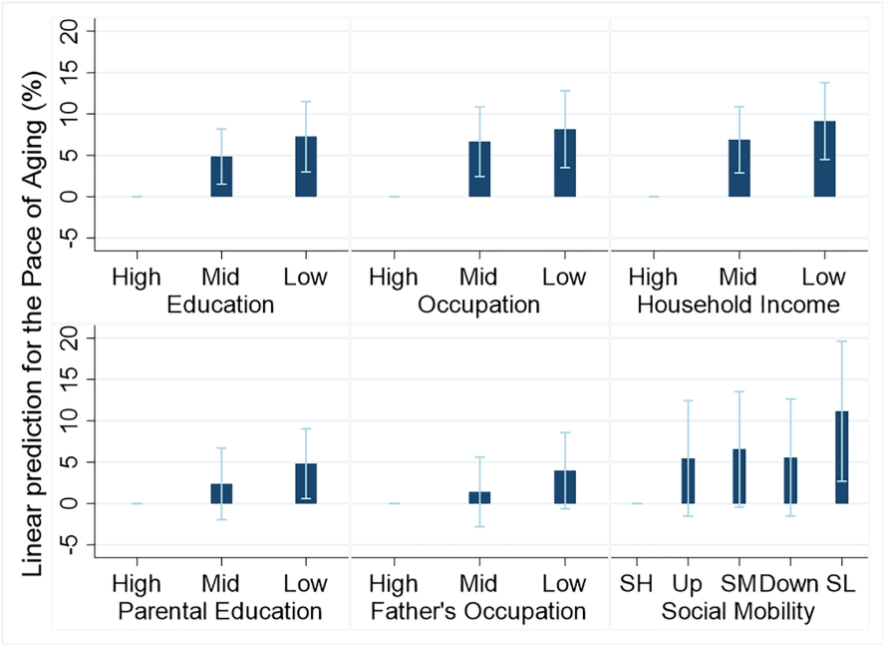
**Graded associations between life course socioecomomic conditions and the Pace of Aging.** Linear regression models adjusted for chronological age and sex. Bar plots indicate coefficients and 95% confidence intervals for each level of the socioeconomic conditions. Abbreviations: SH: stable-high; Up: upward; SM: stable-mid; Down: downward; SL: stable-low.

We used data from 2475 men and 2834 women (aged 35–75 years (mean 52)) in the Swiss population-based cohort CoLaus|PsyCoLaus to test if life course socioeconomic conditions were related to cohort members’ Pace of Aging [5]. Pace of Aging was calculated using three repeat assessments of 12 biomarkers over 11 years. Biomarkers reflected multiple body systems and included mean arterial pressure, glucose, triglycerides, total cholesterol, high-density lipoprotein, BMI, percent body fat, c-reactive protein, creatinine clearance, uric acid, alkaline phosphatase, and gamma-glutamyltransferase. Growth models estimated participants’ personal slopes (change in each biomarker per year), which were aggregated to create a Pace of Aging score. Compared to cohort members who experienced disadvantaged socioeconomic conditions across the life course (low occupational position level of father in childhood and of participant in adulthood), those who experienced consistently advantaged conditions (high occupational position level of father in childhood and of participant in adulthood) aged 10% slower over 11 years follow-up (Figure 1). There was a trend suggesting that those who achieved upward social mobility had slower Pace of Aging as compared with those who experienced persistent socioeconomic disadvantage across the life course. Associations were attenuated but largely persisted when taking into account major health behavior risk factors for chronic disease, including smoking, physical inactivity, and heavy alcohol consumption. The results were unchanged when dropping each biomarker in turn from the Pace of Aging score, suggesting that the findings were not determined by any single biomarker. Associations were also apparent in older age groups (65 years and above), but only for social mobility. Our findings indicate that socioeconomic inequalities contribute to inequalities in the Pace of Aging, partly through differences in health behaviors.

Our findings converge with those of the Dunedin Study investigators, who found that lower parental occupation predicted a faster Pace of Aging in a sample of younger adults [4]. Among older adults, some previous studies found little or no impact of socioeconomic conditions on the rate of physiological decline in individual biomarkers, such as muscle strength and lung function. Combining information from multiple aging-related biomarkers may provide more power to detect risk and protective factors for the rate of physiological decline. However, measuring the Pace of Aging requires data from multiple clinical and biological assays taken over many years, and methodological issues can arise when combining this information into a single index. In response to this challenge, the Dunedin cohort has since developed a DNA-methylation biomarker of the Pace of Aging using machine-learning tools [6]. The advent of omics approaches, such as that used in the Dunedin cohort, has made it possible to quantify thousands of epigenetic marks, transcripts, proteins and metabolites from a single blood draw; opening up the possibility to more accurately assess an individual’s health state for observational studies and geroprotective interventions [7].

Socioeconomic inequalities in the Pace of Aging could be attributed to multiple mechanisms that accumulate across the life course, including differences in access to health care and health-promoting leisure activities, inadequate nutrition, psychosocial stress, and exposure to environmental hazards, as well as direct biological processes relevant to health and longevity. Intervention to promote upward socioeconomic mobility, as well as addressing associated health behaviors, may help slow the pace of aging and increase healthy lifespan for socioeconomically disadvantaged individuals. Interventions to slow aging in at risk individuals may be most effective when delivered by midlife, before aging-related diseases become established.
